# A Very Complete Hysterectomy

**Published:** 1898-03-26

**Authors:** 


### A VERY COMPLETE HYSTERECTOMY.

A recently-suggested method of completely removing the uterus is that described by Chalot, which, among other things, aims at avoiding risk of accidentally wounding the ureters, a danger which is often very real, by transplanting them. The points in this operation are that the whole of the diseased tissue is removed in one mass by incisions wide of the new growth, and that the affected glands are excised. The patient is placed in the Trendelenburg position, a cœliotomy is performed, the internal iliac arteries are tied and divided, the ureters are transplanted into the rectum or the bladder, and the uterus and its appendages are removed. This is said to be a complete operation; but we think it would be fair to hazard the conjecture that it will not be long before someone goes still further. Clearly, if the right kidney were excised and the left ureter implanted into the colon, and a colotomy performed, then the surgeon would have the pelvis all to himself. That would be something like a "complete" operation!

